# Efficacy and safety of apatinib or anlotinib combined with PD-1 inhibitors-based therapy as subsequent-line treatment for recurrent or metastatic nasopharyngeal carcinoma: a real-world retrospective study

**DOI:** 10.3389/fonc.2025.1624286

**Published:** 2025-11-06

**Authors:** Jingjing Gao, Jingshu Xu, Yiyue He, Dan Zong, Tong Jin, Xia He

**Affiliations:** 1 The Affiliated Cancer Hospital of Nanjing Medical University & Jiangsu Cancer Hospital & Jiangsu Institute of Cancer Research, Nanjing, Jiangsu, China; 2 The Fourth School of Clinical Medicine, Nanjing Medical University, Nanjing, Jiangsu, China; 3 Postgraduate College, Xuzhou Medical University, Xuzhou, China

**Keywords:** nasopharyngeal carcinoma, recurrence, metastasis, immune checkpoint inhibitors, anti-angiogenic agent

## Abstract

**Background:**

Recurrent or metastatic nasopharyngeal carcinoma (R/M NPC) that progresses following first-line treatment often ends up with a poor prognosis, and no standard regimens have been established universally. Preclinical studies have suggested that combining vascular endothelial growth factor (VEGF) inhibitors with immune checkpoint inhibitors (ICIs) may exert synergistic antitumor effects. This real-world study aimed to evaluate the efficacy and safety of programmed death-1 (PD-1) inhibitors plus either apatinib or anlotinib, with or without chemotherapy, as a subsequent-line treatment in patients with R/M NPC.

**Methods:**

Between January 1, 2018, and December 12, 2024, a total of 154 patients with R/M NPC were included and treated with various modes of combinations (ITC, IT, IC, I). Among them, 65 received apatinib or anlotinib plus PD-1 inhibitors (ITC+ IT, combination group), and 89 did not receive the addition of apatinib or anlotinib (IC+I, non-combination group). The primary endpoint was progression-free survival (PFS); the secondary endpoints included overall survival (OS), objective response rate (ORR), disease control rate (DCR), and treatment-related adverse events (TRAEs).

**Results:**

As of February 28, 2025, the median follow-up duration was 28.7 months (range 1.3-62.7 months). Compared with the non-combination group, the combination group showed significantly prolonged PFS (20.8 vs. 8.2 months; HR: 0.46, 95% CI: 0.32–0.69; P < 0.001) and OS (34.7 vs. 23.6 months; HR: 0.58, 95% CI: 0.35–0.96; P = 0.042). The combination group also demonstrated higher ORR (47.0% vs. 31.5%; P = 0.041) and DCR (90.8% vs. 82.0%; P = 0.126). The overall incidence of TRAEs was slightly higher in the combination group (96.9% vs. 93.3%; P = 0.599). No treatment-related deaths were reported in either group.

**Conclusion:**

In patients with R/M NPC that progressed after first-line therapy, the combination of anti-angiogenic agents (apatinib or anlotinib) with PD-1 inhibitors based therapy demonstrated a promising antitumor efficacy and an acceptable safety profile. These findings were consistent even among patients from non-endemic regions.

## Introduction

Nasopharyngeal carcinoma (NPC), a malignant tumor arising from the nasopharyngeal epithelium, is classified as undifferentiated non-keratinizing carcinoma in approximately 95% of cases. NPC is strongly associated with Epstein-Barr virus (EBV) infection (1). Globally, NPC accounts for an estimated 133,000 new cases and approximately 80,000 deaths annually, representing 0.7% of all the cancer-related mortality (2). The incidence of NPC elevates markedly in Southern China, Southeast Asia, North Africa, and the Middle East, compared to the global average (3). Due to its deep anatomical location and early non-symptoms, NPC has already progressed into a locally advanced stage in approximately 70% of patients at diagnosis. Subsequent to initial treatments, around 30% of patients still experience local recurrence or distant metastasis, a setting in which conventional salvage chemotherapy just provides limited survival benefit, with a median OS ranging from 10 to 20 months (4, 5).

In 2021, three pivotal multicenter phase III randomized controlled trials—CAPTAIN-1st, JUPITER-02, and RATIONALE-309—demonstrated that the combination of PD-1 inhibitors with chemotherapy significantly improved the OS in patients with R/M NPC receiving first-line therapy (6–8). However, no consensus has reached to propose the optimal treatment strategy for patients who experience disease progression following the first-line therapy. PD-1 inhibitors have been evaluated as a monotherapy or a combiner with chemotherapy in subsequent-line treatments, survival outcomes remain suboptimal (9). Accordingly, novel therapeutic strategies are urgently needed.

Apatinib and anlotinib are small-molecule tyrosine kinase inhibitors (TKIs) that exert antitumor effects by selectively silencing vascular endothelial growth factor (VEGF) signaling. VEGF has been shown to suppress the activation of tumor-associated endothelial cells and downregulate endothelial cell-selective adhesion molecule (ESAM), a key mediator of leukocyte adhesion and transendothelial migration. The downregulation of ESAM impairs antitumor immune responses, thereby facilitating immune evasion (10, 11). Anti-angiogenic agents not only inhibit tumor proliferation by modulating immune cell activity and remodeling the immunosuppressive tumor microenvironment, but also enhance immune surveillance by constraining regulatory T cell expansion and promoting the infiltration of immune effector cells into the tumor (12). Moreover, PD-1 inhibitors can suppress VEGF expression, thereby attenuating tumor-associated angiogenesis and further inhibiting tumor progression and metastasis (13). This synergistic interaction between anti-angiogenic therapy and immune checkpoint blockade has been validated in dealing with multiple solid tumors, including cervical, pancreatic, and gastric cancers (14–16).

Beyond these molecular mechanisms, tumors can also been conceptualized as dynamic pathological ecosystems, where cancer cells act as invasive species that interact, compete, and co-evolve with their microenvironment. In NPC, the tumor microenvironment (TME) is composed of malignant cells, stromal elements, vascular networks, and immune cell populations, together forming a complex ecological community. Through reciprocal interactions, tumor cells continuously adapt to evolve, contributing to tumor progression and therapeutic resistance. On this basis, a combination of immune checkpoint inhibitors with anti-angiogenic agents is hypothesized to achieve dual benefits: restoring immune activity while breaking tumor vasculature, thereby facilitating immune cell infiltration and ultimately improving treatment efficacy (17, 18).

Increasing evidence has underscored notable differences in the pathogenesis and tumor immune microenvironment (TIME) of NPC between patients from endemic and non-endemic regions. In endemic regions, NPC is predominantly classified as non-keratinizing undifferentiated carcinoma, and is strongly associated with EBV infection (19). Persistent EBV infection induces chronic inflammatory responses, leading to a tumor microenvironment enriched with regulatory T cells (Tregs), myeloid-derived suppressor cells (MDSCs), and elevated PD-L1 expression, collectively establishing a profoundly immunosuppressive and exhausted immune state. Consequently, patients from endemic regions tend to exhibit higher response rates and more durable clinical benefits, when treated with PD-1/PD-L1 immune checkpoint inhibitors. In contrast, NPC arising in non-endemic regions is more frequently driven by genomic alterations, such as TP53 mutations and CDKN2A inactivation, and is less commonly associated with EBV infection (20). Accordingly, the TIME in non-endemic NPC is typically characterized by a reduced immune cell infiltration and a low PD-L1 expression, reflecting a “cold tumor” phenotype (21). Under such immunologically quiescent conditions, a monotherapy with immune checkpoint inhibitors often fails to elicit a robust antitumor immune response, leading to a lower response rate and a limited clinical efficacy.

In the light of these regional disparities, we conducted a retrospective analysis of 154 patients with R/M NPC from a non-endemic region. This real-world study aimed to evaluate the efficacy and safety of incorporating apatinib or anlotinib into PD-1 inhibitors-based regimens, with or without chemotherapy.

## Materials and methods

### Inclusion and exclusion criteria

Inclusion criteria were as follows: (1) Histologically confirmed R/M NPC; (2) Disease progression following at least first-line of systemic therapy for recurrent or metastatic disease; (3) Eastern Cooperative Oncology Group (ECOG) performance status of 0-1; (4) Ages between 15 and 80 years; (5) At least one measurable lesion according to the Response Evaluation Criteria in Solid Tumors, version 1.1 (RECIST 1.1); (6) Receipt of at least two cycles of PD-1 inhibitor therapy, administered either as monotherapy or in combination with apatinib or anlotinib.

Exclusion criteria included the following: (1) History of other malignancies; (2) Inability to evaluate treatment efficacy; (3) Receipt of fewer than two cycles of immunotherapy; (4) Receipt of first-line therapy after disease progression; (5) Prior exposure to VEGF inhibitors other than apatinib or anlotinib.

### Treatment regimens

Chemotherapy agents: paclitaxel, 260 mg/m²on day 1, intravenous infusion (IV); docetaxel, 75 mg/m²on day 1, IV; gemcitabine, 1 g/m²on days 1 and 8, IV; cisplatin, 70 mg/m²on day 1, IV; 5-fluorouracil, 600 mg/m²on day 1, IV; capecitabine, 650 mg/m², orally twice daily.
PD-1 inhibitors: camrelizumab, tislelizumab, and sintilimab, 200 mg on day 1, IV; toripalimab, 240 mg on day 1, IV.
Anti-angiogenic agents: apatinib, 250 mg orally, once daily; anlotinib, 12 mg orally, once daily on days 1–14 in each 21-day cycle.

One regimen was administered every 3 weeks and continued until disease progression, unacceptable toxicity, or necessary treatment modification or discontinuation decided by the physician.

### Endpoints and assessment

Objective response rate (ORR) was defined as the proportion of patients achieving a complete response (CR) or partial response (PR). Disease control rate (DCR) was defined as the proportion of patients achieving CR, PR, or stable disease (SD). OS was calculated from the date of treatment initiation to the date of death from any cause. Progression-free survival (PFS) was calculated from treatment initiation to the first documented disease progression or death. Treatment response was evaluated according to the RECIST v1.1, after 2–3 treatment cycles. Adverse events (AEs) were assessed and graded in accordance with the Common Terminology Criteria for Adverse Events, version 5.0 (CTCAE v5.0). The primary endpoint was PFS. The secondary endpoints included OS, ORR, DCR, and the incidence of treatment-related AEs.

### Statistical analysis

Baseline characteristics, tumor response rates, and AEs were compared using the chi-square (χ²) test. The Kaplan-Meier analysis was employed to estimate survival outcomes, and differences between groups were assessed using the log-rank test. Prognostic factors and subgroup analyses were evaluated using the Cox proportional hazards regression model. All statistical analyses were performed using R software and GraphPad Prism version 10.2.3. P-value <0.05 was considered statistically significant.

## Results

### Patient characteristics

A total of 154 patients with R/M NPC progressing after at least one line of salvage therapy were included and categorized into four treatment groups. The median follow-up duration was 28.7 months (range 1.3–62.7 months), during which 103 patients (66.9%) experienced disease progression. Among these, 31 patients (30.1%) showed locoregional recurrence, while 72 (69.9%) showed distant metastases. As of the data cutoff (February 28, 2025), 63 patients (40.9%) had died.

**Figure 1:**
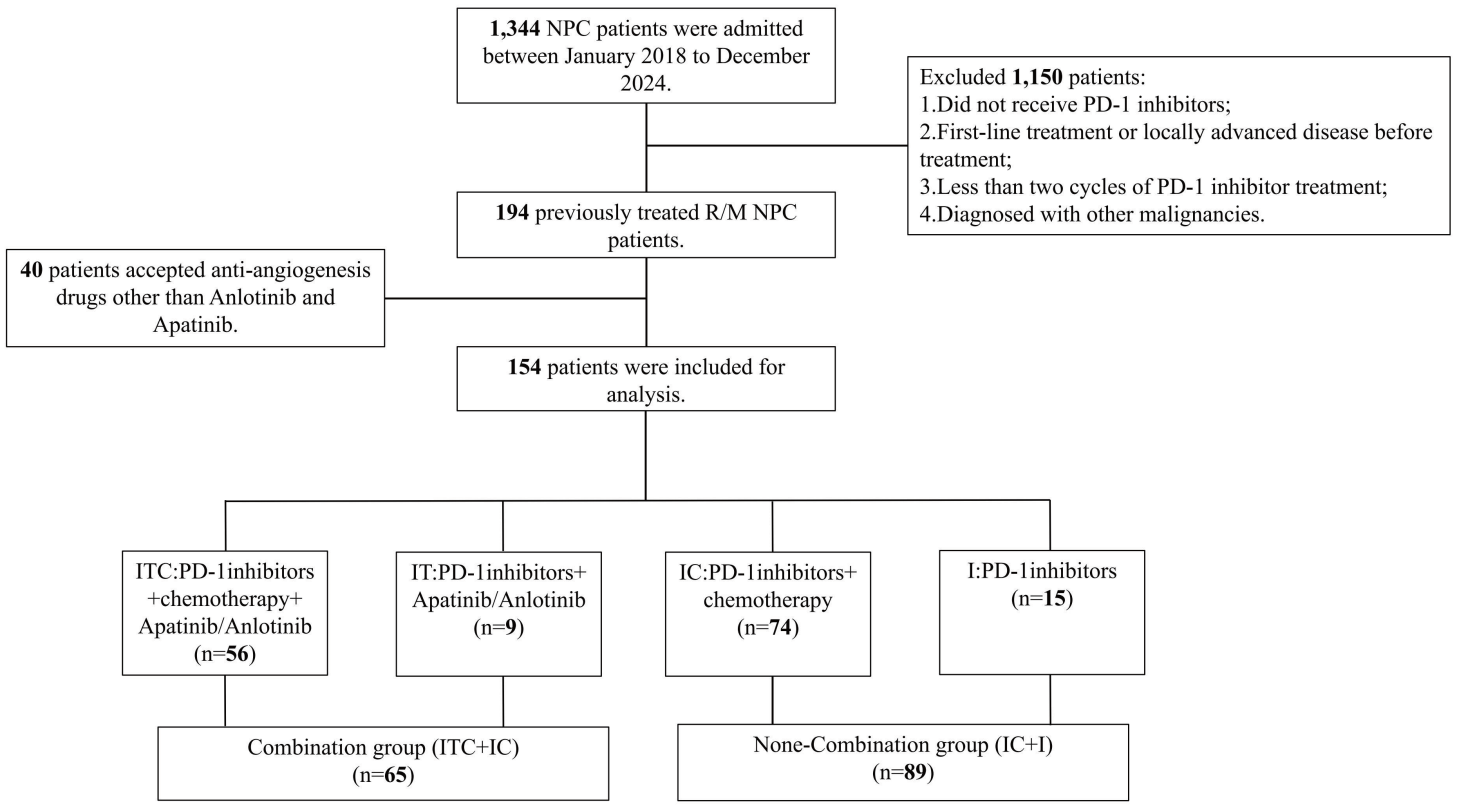
Detailed patient selection process. ITC, PD-1 inhibitors plus apatinib or anlotinib and chemotherapy; IT, PD-1 inhibitors plus apatinib or anlotinib; IC, PD-1 inhibitors plus chemotherapy.

Of the enrolled patients, 56 received the ITC regimen (PD-1 inhibitor + chemotherapy + apatinib/anlotinib), 74 received the IC regimen (PD-1 inhibitor + chemotherapy), 15 received the I regimen (PD-1 inhibitor monotherapy), and 9 received the IT regimen (PD-1 inhibitor + apatinib/anlotinib). Considering the relatively small sample sizes of the IT and I subgroups, to evaluate the impact of incorporating anti-angiogenic agents on clinical outcomes, the patients receiving the ITC and IT regimens were grouped into the combination arm (n = 65), while those receiving the IC and I regimens into the non-combination arm (n = 89). The patient selection process is detailed in **Figure 1**. The median age of the study population was 46 years (range 18–71 years), with a male-to-female ratio of 3.5:1. Within the combination arm, 13 patients (20.0%) received apatinib and 52 (80.0%) received anlotinib. Regarding PD-1 inhibitor administration, 47 patients (30.5%) were treated with camrelizumab, 41 (26.6%) with toripalimab, 38 (24.7%) with tislelizumab, and 28 (18.2%) with sintilimab. Baseline clinical and demographic characteristics are summarized in **Table 1**; **Supplementary Table S1**, with no significant differences observed between the two arms or two major treatment subgroups (ITC subgroup and IC subgroup).

**Table 1:** Baseline characteristics of the two groups.

Characteristic	Combination group (N = 65)	None-combination group (N = 89)	Total (N=154)	P-value
Age				
≤50	33 (50.8%)	39 (43.8%)	72 (46.8%)	0.695
>50	32 (49.2%)	50 (56.2%)	82 (53.2%)	
Gender				
Male	51 (78.5%)	69 (77.5%)	120 (77.9%)	0.991
Female	14 (21.5%)	20 (22.5%)	34 (22.1%)	
Smoking				
No	62 (95.4%)	80 (89.9%)	142 (92.2%)	0.454
Yes	3 (4.6%)	9 (10.1%)	12 (7.8%)	
Cancer family history				
No	64 (98.5%)	85 (95.5%)	149 (96.8%)	0.593
Yes	1 (1.5%)	4 (4.5%)	5 (3.2%)	
Underlying disease history				
No	49 (75.4%)	68 (76.4%)	117 (76.0%)	0.989
Yes	16 (24.6%)	21 (23.6%)	37 (24.0%)	
BMI				
<18	6 (9.2%)	7 (7.9%)	13 (8.4%)	0.932
18-24	36 (55.4%)	44 (49.4%)	80 (51.9%)	
>24	23 (35.4%)	38 (42.7%)	61 (39.6%)	
Disease status				
Recurrent	22 (33.8%)	21 (23.6%)	43 (27.9%)	0.375
Metastatic	43 (66.2%)	68 (76.4%)	111 (72.1%)	
Treatment lines				
2	44 (67.7%)	67 (75.3%)	111 (72.1%)	0.584
≥3	21 (32.3%)	22 (24.7%)	43 (27.9%)	
Liver metastasis				
No	45 (69.2%)	63 (70.8%)	108 (70.1%)	0.979
Yes	20 (30.8%)	26 (29.2%)	46 (29.9%)	
EBV DNA level				
Negative	26 (40.0%)	41 (46.1%)	67 (43.5%)	0.755
Positive	39 (60.0%)	48 (53.9%)	87 (56.5%)	
Number of metastatic sites				
0-3	31 (47.7%)	36 (40.4%)	67 (43.5%)	0.670
>3	34 (52.3%)	53 (59.6%)	87 (56.5%)	
Previous treatment				
Platinum based therapy	45 (69.2%)	67 (75.3%)	112 (72.7%)	0.707
PD-1 inhibitors based therapy	20 (30.8%)	22 (24.7%)	42 (27.3%)	
Chemotherapy				
No	9 (13.8%)	15 (16.9%)	24 (15.6%)	0.879
Yes	56 (86.2%)	74 (83.1%)	130 (84.4%)	
Local radiotherapy				
No	23 (35.4%)	41 (46.1%)	64 (41.6%)	0.414
Yes	42 (64.6%)	48 (53.9%)	90 (58.4%)	
Pathological classification (Squamous-cell carcinoma)				
Undiffrentiation	35 (53.8%)	44 (49.4%)	79 (51.3%)	0.689
Lowdifferentiation	30 (46.2%)	45 (50.6%)	75 (48.7%)	
Clinical stage				
III stage	10 (15.4%)	9 (10.1%)	19 (12.3%)	0.326
IV stage	55 (84.6%)	80 (89.9%)	135 (87.7%)	

BMI, body mass index; EBV, Epstein–Barr virus.

### Tumor response

Among the 65 patients in the combination arm, 4 (6.2%) achieved CR, 27 (41.5%) achieved PR, 28 (43.1%) had SD, and 6 (9.2%) experienced PD. The ORR in the combination group was 69.1%, significantly higher than that observed in the non-combination group (49.5%) (P = 0.011). However, no significant difference in DCR was noted between the two groups (90.8% vs. 82.0%, P = 0.837). Detailed results are presented in **Table 2**. In addition, no statistically significant differences in ORR (48.2% vs. 31.7%, P = 0.139) and DCR (92.9% vs. 81.1%, P = 0.157) were observed between the ITC subgroup and the IC subgroup. (**Supplementary Table S2**).

**Table 2:** Tumor response in the two groups.

Response	Combination group (N = 65)	None-combination group (N = 89)	Total (N = 154)	P-value
CR	4 (6.2%)	2 (2.2%)	6 (3.9%)	
PR	27 (41.5%)	26 (29.2%)	53 (34.4%)	
SD	28 (43.1%)	45 (50.6%)	73 (47.4%)	
PD	6 (9.2%)	16 (18.0%)	22 (14.3%)	
ORR	31 (47.7%)	28 (31.5%)	59 (38.3%)	**0.041**
DCR	59 (90.8%)	73 (82.0%)	132 (85.7%)	0.126

CR, complete response; PR, partial response; SD, stable disease; PD, progressive disease; ORR, objective response rate; DCR, disease control rate. *P-value in bold indicate statistical significance (P < 0.05).

### Survival outcomes

**Figure 2:**
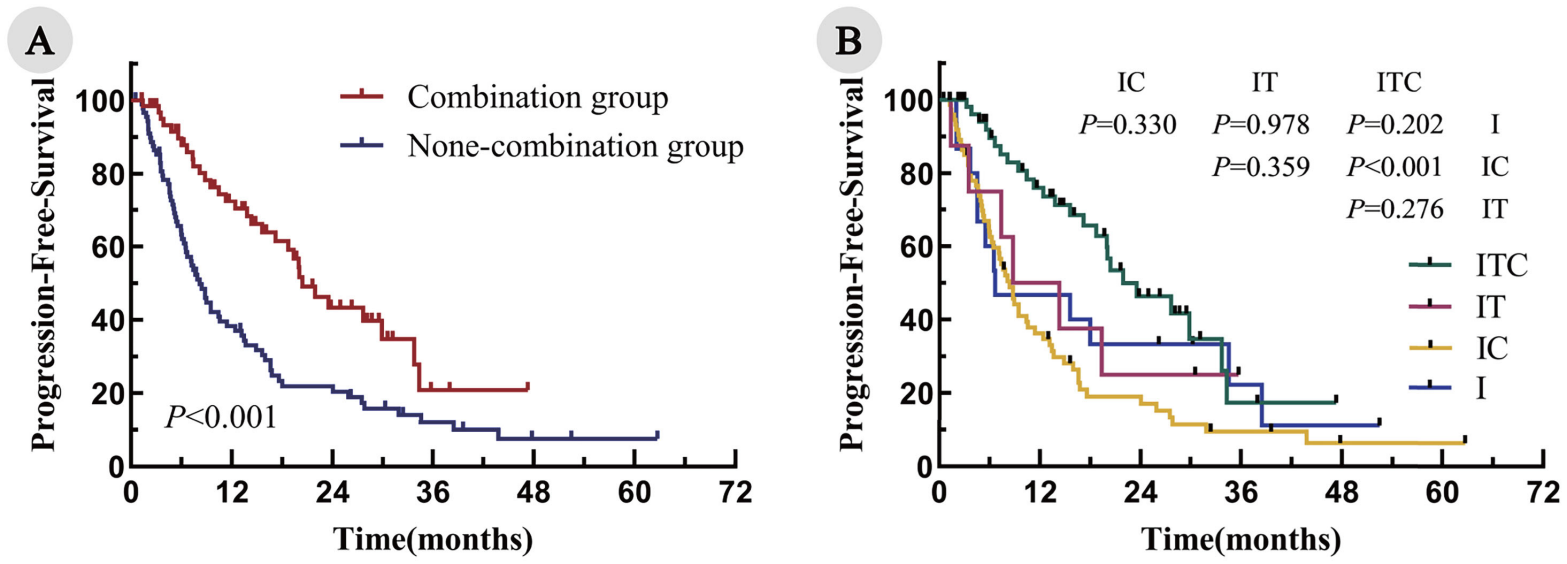
Kaplan–Meier curves for PFS in all enrolled patients. **(A)** Combination group vs. non-combination group; **(B)** ITC, IT, IC, and I cohorts.

**Figure 3:**
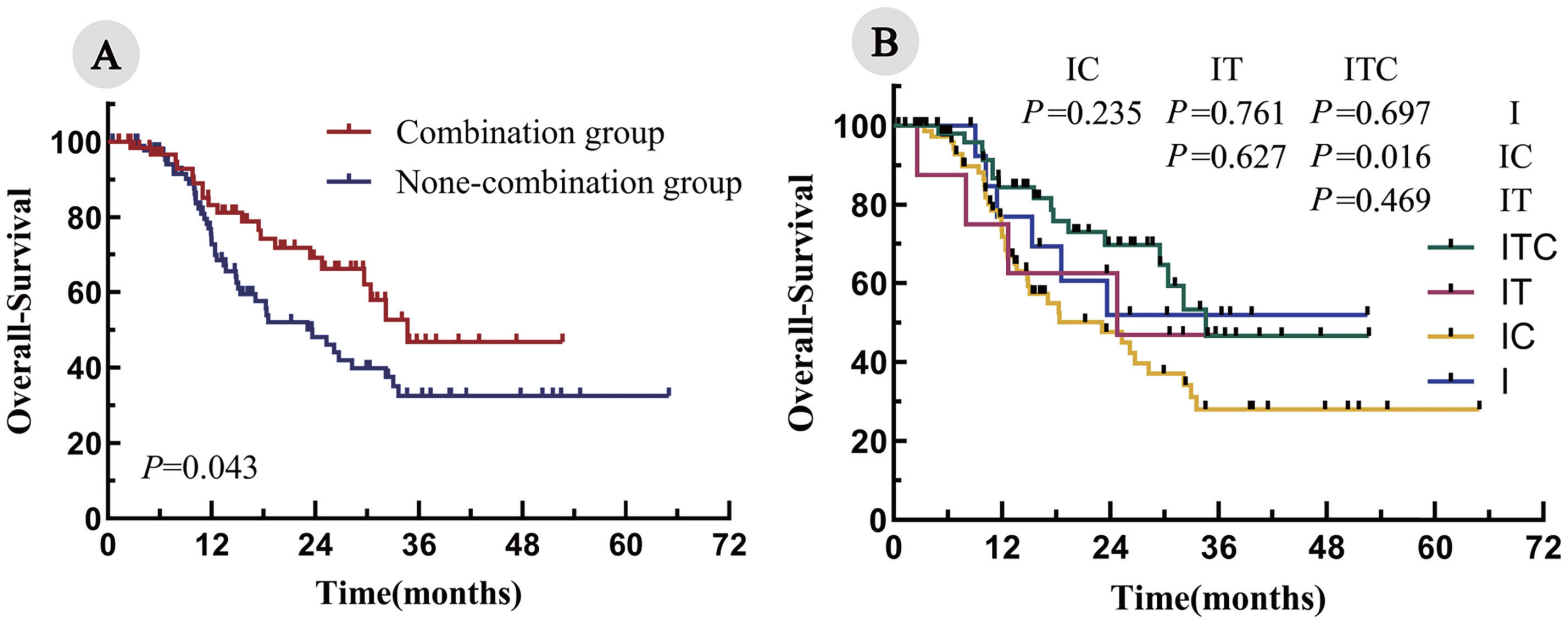
Kaplan–Meier curves for OS in all enrolled patients. **(A)** Combination group vs. none-combination group; **(B)** ITC, IT, IC, and I cohorts.

The median follow-up duration was 28.7 months. The median PFS was significantly longer in the combination group, compared with the non-combination group (20.8 months vs. 8.2 months; P < 0.001; **Figure 2A**). Among the four cohorts, the median PFS was as follows: ITC, 20.8 months; IT, 11.6 months; IC, 8.4 months; and I, 6.7 months. Notably, the ITC cohort demonstrated a statistically significant PFS advantage over the IC cohorts (P < 0.001; **Figure 2B**). Similarly, the OS was significantly prolonged in the combination arm, compared with the non-combination arm (34.7 months vs. 23.6 months; P = 0.043; **Figure 3A**). When comparing individual cohorts, a statistically significant difference in OS was observed only between the ITC and IC cohorts (34.7 months vs. 23.1 months; P = 0.016), while no significant differences were identified among the remaining cohorts (**Figure 3B**).

The multivariate Cox regression analysis, adjusted for relevant prognostic factors, identified combination therapy as an independent predictor of improved PFS (hazard ratio [HR] = 0.391; P < 0.001). Additional favorable prognostic indicators included serum lactate dehydrogenase (LDH) ≤240 U/L, prognostic nutritional index (PNI) >47.2, 0–3 metastatic sites, and receipt of more than six treatment cycles (**Table 3**; **Supplementary Figure S1**). However, OS did not demonstrate independent prognostic value (hazard ratio [HR] = 0.823; P = 0.522) (**Table 4**). The analysis of the ITC and IC subgroups also revealed similar results (**Supplementary Tables S3**, **S4**).

**Table 3:** Univariate and multivariate Cox regression analysis of prognostic factors in all enrolled patients (PFS).

Variable	Univariate analysis		Multivariable analysis	
	HR (95%CI)	P-value	HR (95%CI)	P-value
Gender (female vs male)	0.965 (0.600-1.552)	0.884		
Age (>50 vs ≤50)	1.111 (0.753-1.640)	0.596		
EBV DNA level (positive vs negative)	1.968 (1.311-2.953)	**0.001**	1.408 (0.852-2.329)	0.182
Anemia (yes vs no)	1.862 (1.262-2.747)	**0.002**	0.955 (0.572-1.596)	0.862
PLT (>110 vs ≤110)	0.566 (0.321-1.000)	0.050		
ALB (>41.3 vs ≤41.3)	0.450 (0.289-0.700)	**<0.001**	0.739 (0.377-1.451)	0.380
LDH (>240 vs ≤240)	2.905 (1.814-4.654)	**<0.001**	4.013 (1.676-9.611)	**0.002**
PLR (>94.37 vs ≤94.37)	0.531 (0.319-0.884)	**0.015**	0.728 (0.392-1.351)	0.314
NLR (>5.5 vs ≤5.5)	1.765 (1.153-2.701)	**0.009**	1.365 (0.792-2.355)	0.263
LAR (>5.43 vs ≤5.43)	3.156 (1.987-5.013)	**<0.001**	0.425 (0.158-1.145)	0.091
PNI (>47.2 vs ≤47.2)	0.435 (0.289-0.653)	**<0.001**	0.479 (0.244-0.941)	**0.033**
Treatment lines (≥3 vs 2)	1.524 (1.011-2.297)	**0.044**	1.393 (0.860-2.255)	0.178
Distant metastasis (Yes vs No)	1.303 (0.838-2.025)	0.240		
Liver metastasis (Yes vs No)	1.843 (1.224-2.773)	**0.003**	1.373 (0.821-2.294)	0.227
Bone metastasis (Yes vs No)	1.303 (0.882-1.924)	0.183		
Lung metastasis (Yes vs No)	0.844 (0.546-1.306)	0.447		
Number of metastatic sites (>3 vs 0-3)	1.864 (1.237-2.809)	**0.003**	1.681 (1.003-2.818)	0.049
Treatment cycles (>6 vs 2-6)	0.365 (0.242-0.549)	**<0.001**	0.279 (0.171-0.456)	**<0.001**
Combination treatment (Yes vs No)	0.448 (0.293-0.685)	**<0.001**	0.374 (0.233-0.601)	**<0.001**
Local radiotherapy (Yes vs No)	0.675 (0.457-0.998)	**0.049**	0.735 (0.474-1.141)	0.170
Previous treatment (PD-1 inhibitors based therapy vs Platinum based therapy)	1.687 (1.019-2.568)	**0.015**	1.735 (1.051-2.864)	**0.031**
Pathological classification (Undiffrentiation vs Lowdifferentiation)	0.859 (0.579-1.275)	0.452		
Clinical stage (IV vs III)	1.538 (0.798-2.963)	0.199		

HR, hazard ratio; CI, confidence interval; EBV, Epstein-Barr virus; PLT, Platelet; ALB, Albumin; LDH, Lactate dehydrogenase; PLR, platelet to lymphocyte ratio; NLR, neutrophil to lymphocyte ratio; LAR, Lactate dehydrogenase to Albumin ratio; PNI, Prognostic nutritional index. *P-value in bold indicate statistical significance (P < 0.05).

**Table 4:** Univariate and multivariate Cox regression analysis of prognostic factors in all enrolled patients (OS).

Variable	Univariate analysis		Multivariable analysis	
	HR (95%CI)	P-value	HR (95%CI)	P-value
Gender (female vs male)	1.155 (0.646-2.062)	0.627		
Age (>50 vs ≤50)	1.130 (0.688-1.857)	0.628		
EBV DNA level (positive vs negative)	2.759 (1.585-4.80)	**<0.001**	1.536 (0.798-2.955)	0.199
Anemia (yes vs no)	2.107 (1.281-3.466)	**0.002**	0.767 (0.404-1.457)	0.418
PLT (>110 vs ≤110)	0.567 (0.311-1.391)	0.272		
ALB (>41.3 vs ≤41.3)	0.366 (0.215-0.624)	**<0.001**	1.180 (0.538-2.587)	0.769
LDH (>240 vs ≤240)	2.783 (1.670-4.931)	**<0.001**	0.887 (0.296-2.656)	0.830
PLR (>94.37 vs ≤94.37)	0.511 (0.273-0.959)	**0.037**	0.461 (0.221-0.965)	**0.040**
NLR (>5.5 vs ≤5.5)	1.962 (1.172-3.283)	**0.010**	1.028 (0.526-2.010)	0.935
LAR (>5.43 vs ≤5.43)	4.201 (2.462-7.165)	**<0.001**	2.260 (0.712-7.173)	0.166
PNI (>47.2 vs ≤47.2)	0.300 (0.182-0.493)	**<0.001**	0.290 (0.134-0.627)	**0.002**
Treatment lines (≥3 vs 2)	1.566 (0.936-2.622)	0.088		
Distant metastasis (Yes vs No)	1.452 (0.801-2.630)	0.219		
Liver metastasis (Yes vs No)	2.242 (1.355-3.711)	**0.002**	1.256 (0.651-2.423)	0.496
Bone metastasis (Yes vs No)	1.250 (0.762-2.053)	0.377		
Lung metastasis (Yes vs No)	1.282 (0.763-2.155)	0.348		
Number of metastatic sites (>3 vs 0-3)	2.586 (1.464-4.568)	**0.001**	2.094 (1.086-4.036)	**0.027**
Treatment cycles (>6 vs 2-6)	0.310 (0.181-0.532)	**<0.001**	0.268 (0.140-0.514)	**<0.001**
Combination treatment (Yes vs No)	0.580 (0.341-0.987)	**0.045**	0.823 (0.454-1.493)	0.522
Local radiotherapy (Yes vs No)	0.609 (0.372-0.999)	0.050		
Previous treatment (PD-1 inhibitors based therapy vs Platinum based therapy)	1.602 (0.943-2.722)	0.082		
Pathological classification (Undiffrentiation vs Lowdifferentiation)	0.970 (0.588-1.598)	0.904		
Clinical stage (IV vs III)	1.243 (0.566-2.730)	0.588		

HR, hazard ratio; CI, confidence interval; EBV, Epstein-Barr virus; PLT, Platelet; ALB, Albumin; LDH, Lactate dehydrogenase; PLR, platelet to lymphocyte ratio; NLR, neutrophil to lymphocyte ratio; LAR, Lactate dehydrogenase to Albumin ratio; PNI, Prognostic nutritional index. *P-value in bold indicate statistical significance (P < 0.05).

**Figure 4:**
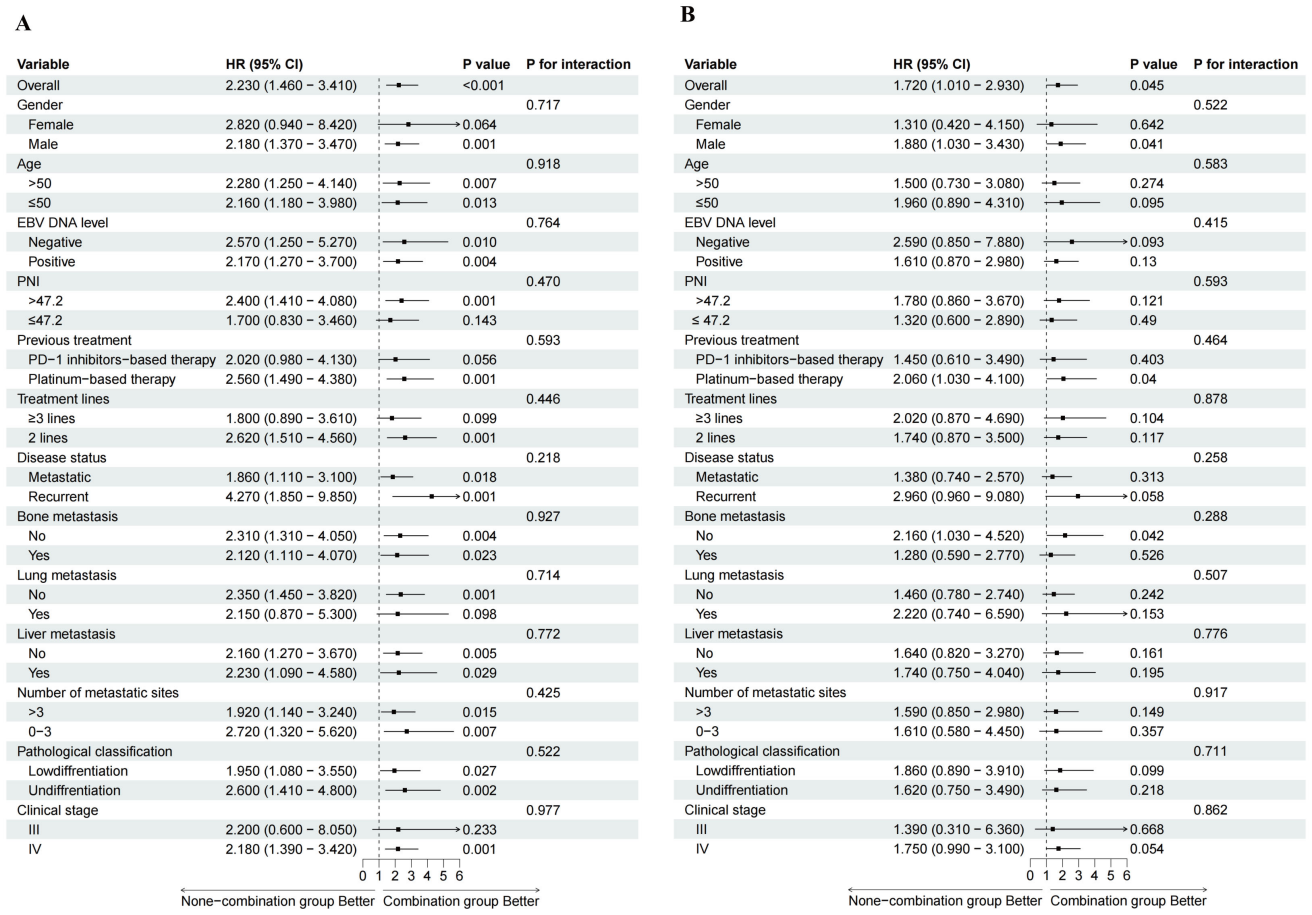
Forest plot of subgroup analyses. HR, hazard ratio; CI, confidence interval; EBV, Epstein-Barr virus; PNI, Prognostic nutritional index. **(A)** subgroup analyses for PFS; **(B)** subgroup analyses for OS.

The subgroup analysis further demonstrated that the combination group was associated with the prolonged PFS and OS across all subgroups, with the most pronounced benefit observed in male patients or an absence of liver metastases (P < 0.05). No significant interaction effects were identified among subgroups (**Figures 4A, B**; **Supplementary Figures S2A, B**). In addition, the stratified analysis suggested that, across all treatment subgroups, patients with prior PD-1 inhibitor exposure had shorter PFS and OS compared with PD-1 naïve patients, as specifically shown in **Supplementary Figure S3**.

### AEs

As of the last follow-up, no treatment-related deaths were reported. The most frequently observed AEs in both arms involved anemia (84.6% vs. 82.0%; P = 0.914) and leukopenia (67.7% vs. 64.0%; P = 0.895). The overall incidence of any-grade AEs was slightly higher in the combination group, compared to the non-combination group (96.9% vs. 93.3%; P = 0.599), so was the incidence of grade ≥3 AEs (38.5% vs. 30.3%; P = 0.574). The combination group exhibited higher incidences of nasopharyngeal necrosis (6.2% vs. 4.5%; P = 0.900) and hypertension (29.2% vs. 15.7%; P = 0.131), although these differences were not statistically significant. Conversely, the combination group demonstrated lower rates of grade ≥3 anemia (3.1% vs. 10.1%; P = 0.246), thrombocytopenia (7.7% vs. 15.7%; P = 0.326), and leukopenia (20.0% vs. 22.5%; P = 0.934) (**Table 5**; **Supplementary Table S5**).

**Table 5:** Treatment-related adverse events in all enrolled patients.

Adverse events	All grades			≥3 grade		
	Combination group(N = 65)	None-combination group(N = 89)	P-value	Combination group(N = 65)	None-combination group(N = 89)	P-value
Fatigue	28 (43.1%)	28 (31.5%)	0.334	9 (13.8%)	7 (7.9%)	0.486
Nausea	29 (44.6%)	33 (37.1%)	0.642	7 (10.8%)	6 (6.7%)	0.674
Anemia	55 (84.6%)	73 (82.0%)	0.914	2 (3.1%)	9 (10.1%)	0.246
Leukopenia	44 (67.7%)	57 (64.0%)	0.895	13 (20.0%)	20 (22.5%)	0.934
Thrombocytopenia	39 (60.0%)	47 (52.8%)	0.674	5 (7.7%)	14 (15.7%)	0.326
Hypertension	19 (29.2%)	14 (15.7%)	0.131	3 (4.6%)	2 (2.2%)	0.715
ALT elevation	24 (36.9%)	30 (33.7%)	0.918	3 (4.6%)	2 (2.2%)	0.715
AST elevation	17 (26.2%)	29 (32.6%)	0.690	1 (1.5%)	2 (2.2%)	0.952
Hypoalbuminemia	22 (33.8%)	23 (25.8%)	0.559	0	1 (1.1%)	0.692
Rash	4 (6.2%)	2 (2.2%)	0.465	2 (3.1%)	0	0.250
Pneumonia	3 (4.6%)	2 (2.2%)	0.715	0	1 (1.1%)	0.692
Epistaxis	3 (4.6%)	4 (4.5%)	0.999	3 (4.6%)	0	0.123
NP necrosis	4 (6.2%)	4 (4.5%)	0.900	1 (1.5%)	1 (1.1%)	0.975
Cough	5 (7.7%)	2 (2.2%)	0.277	0	0	1.000
Hypothyroidism	23 (35.4%)	43 (48.3%)	0.277	5 (7.7%)	8 (9.0%)	0.960
Total	63 (96.9%)	83 (93.3%)	0.599	25 (38.5%)	27 (30.3%)	0.574

ALT, alanine aminotransferase; AST, aspartate aminotransferase.

## Discussion

To the best of our knowledge, this is the first study to evaluate the efficacy and safety of PD-1 inhibitors combined with apatinib or anlotinib, with or without chemotherapy, in patients with R/M NPC who had experienced disease progression following the first-line therapy in non-endemic regions. Although the introduction of ICIs has improved clinical outcomes in this population, the overall prognosis remains unsatisfactory. Previous studies have reported that PD-1 inhibitor monotherapy yields a median PFS of approximately 1.9 to 6.5 months and an ORR of 21%-26% (22). In this light, new combination strategies are urgently needed to enhance treatment efficacy.

Several clinical trials, conducted in highly endemic regions, have investigated the antitumor efficacy of PD-1 inhibitors combined with anti-angiogenic agents in patients with advanced head and neck squamous cell carcinoma (HNSCC), reporting an ORR ranging from 33.3% to 65.5% and a median PFS between 6.0 and 14.3 months (23–27). In a retrospective analysis, Jiang et al. have compared the efficacy of ICIs in combination with either VEGF/VEGFR or EGFR inhibitors plus chemotherapy in the subsequent-line setting (28). The addition of targeted agents significantly prolongs the median PFS, compared with chemotherapy alone or chemotherapy plus ICIs (19.1 vs. 9.8 months; P < 0.001), consistent with our study findings. Moreover, our data demonstrated a significant improvement in the median OS in the combination group. Prognostic factor analysis further identified hematological markers, such as LDH and PNI, as independent predictors of clinical outcomes. Previous studies have shown that elevated baseline LDH levels are frequently associated with an increased tumor burden and a hypoxic tumor microenvironment (29). Additionally, LDH has been implicated in the activation of oncogenic signaling pathways, tumor metabolism, invasiveness, and immunologic modulation (30). Here, our subgroup analysis revealed that combination therapy provided a greater clinical benefit among male patients, those who were EBV-DNA positive, or those who had previously failed the platinum-based chemotherapy. These findings offer valuable insight for future patient stratification in clinical practice.

In recent years, disease progression has increasingly challenged the efficacy of immunotherapy, underscoring the urgent need to improve clinical outcomes in this setting. The mechanisms underlying the immune resistance to PD-1 inhibitors are multifactorial and remain incompletely understood. On the one hand, tumor cells can evade immune surveillance by downregulating the expression of major histocompatibility complex class I (MHC-I) molecules and other key components of the antigen presentation machinery. On the other hand, an elevated PD-L1 expression on tumor cells facilitates PD-1/PD-L1 binding, thus initiating inhibitory signaling pathways that impair T cell activation and attenuate antitumor immune responses (31, 32). Ding et al. and Yuan et al. have independently assessed the efficacy of camrelizumab in combination with either apatinib or famitinib in patients exhibiting immune-refractory disease, reporting an ORR of approximately 34% (26, 33). In addition, Xiang et al. have shown that the combination of ICIs with targeted therapies yields a significantly higher DCR and a prolonged median PFS, compared with chemotherapy alone (P < 0.001). Although our stratified and subgroup analyses indicated a favorable prognostic trend for the combination group or the ITC subgroup in immune-resistant patients, the unique benefit of this combination strategy for this population could not be confirmed. Future studies with larger sample sizes and prospective designs are warranted to further validate these findings and to provide more robust evidence for precision therapy in immune-resistant patients.

With regard to treatment-related AEs, no significant differences were observed between the two groups, and no treatment-related deaths occurred, indicating that the combination regimen maintains a manageable profile of safety. Myelosuppression, gastrointestinal symptoms, and hypothyroidism were the most commonly reported AEs in both arms. Notably, the incidence of grade ≥3 myelosuppression was lower in the combination group, potentially attributed to dose reductions in platinum-based chemotherapy when used alongside apatinib or anlotinib. However, the combination groups exhibited a higher incidence of severe nasopharyngeal necrosis or epistaxis, compared to the non-combination group. Prior studies have identified radiotherapy doses ≥72 Gy, re-irradiation, locally advanced disease, diabetes mellitus, and smoking history as risk factors for nasopharyngeal necrosis (34–36). Therefore, early identification of high-risk patients and timely clinical intervention during the combination therapy are essential to mitigate the risk of serious complications.

While this study contributes valuable data on the efficacy of combination therapy in R/M NPC patients from non-endemic regions, several limitations should be acknowledged. First, given that all patients were enrolled from a single center, the generalizability of the findings may be limited. Second, this study failed to fully demonstrate an independent advantage of the combination regimen in extending the OS, which may be attributed to the relatively small sample size and insufficient follow-up duration; therefore, larger, multicenter, randomized controlled trials are warranted to validate these results. Third, as this was a retrospective study, inherent heterogeneity could not be fully avoided, particularly in terms of the diversity of chemotherapy regimens and PD-1 inhibitors administered across groups, which also limited our ability to directly determine the impact of specific combination strategies on patient prognosis. Moreover, the relatively small sample sizes of the IT and I subgroups precluded the performance of multi-arm subgroup analyses. Lastly, the subset of patients lacked data on PD-L1 tumor expression, which prevented its inclusion in subgroup analyses and related exploratory assessments.

## Conclusion

The combination of anti-angiogenic agents (apatinib or anlotinib) with PD-1 inhibitors based therapy significantly prolonged the PFS and OS in patients with R/M NPC who had undergone failures in prior therapies, without a notable increase in treatment-related AEs. This combination strategy offers a promising antitumor effect and an acceptable safety in patients with R/M NPC from non-endemic regions, supporting its potential as a subsequent-line therapeutic option.

## Data Availability

The raw data supporting the conclusions of this article will be made available by the authors, without undue reservation.

## Supplementary material

Supplementary Table 1 Baseline characteristics of the two treatment subgroups (ITC and IC). BMI, body mass index; EBV, Epstein–Barr virus.

Supplementary Table 2 Tumor response in the two treatment groups (ITC and IC). CR, complete response; PR, partial response; SD, stable disease; PD, progressive disease; ORR, objective response rate; DCR, disease control rate.

Supplementary Table 3 Univariate and multivariate Cox regression analysis of prognostic factors in ITC and IC subgroups (PFS). HR, hazard ratio; CI, confidence interval; EBV, Epstein-Barr virus; PLT, Platelet; ALB, Albumin; LDH, Lactate dehydrogenase; PLR, platelet to lymphocyte ratio; NLR, neutrophil to lymphocyte ratio; LAR, Lactate dehydrogenase to Albumin ratio; PNI, Prognostic nutritional index.

Supplementary Table 4 Univariate and multivariate Cox regression analysis of prognostic factors in ITC and IC subgroups (OS). HR, hazard ratio; CI, confidence interval; EBV, Epstein-Barr virus; PLT, Platelet; ALB, Albumin; LDH, Lactate dehydrogenase; PLR, platelet to lymphocyte ratio; NLR, neutrophil to lymphocyte ratio; LAR, Lactate dehydrogenase to Albumin ratio; PNI, Prognostic nutritional index.

Supplementary Table 5 Treatment-related adverse events in ITC and IC subgroups. ALT, alanine aminotransferase; AST, aspartate aminotransferase.

Supplementary Figure 1 The optimal cut-off values for hematological markers were determined using the surv_cutpoint function from the survminer package in R, which identifies the most statistically significant threshold based on maximally selected rank statistics. (PLT, Platelet; ALB, Albumin; LDH, Lactate dehydrogenase; PLR, platelet to lymphpcyte ratio; NLR, neutrophil to lymphocyte ratio; LAR, Lactate dehydrogenase to Albumin ratio; PNI, Prognostic nutritional index).

Supplementary Figure 2 Forest plot of subgroup analyses (ITC and IC). HR, hazard ratio; CI, confidence interval; EBV, Epstein-Barr virus; PNI, Prognostic nutritional index. **(A)** subgroup analyses for PFS; **(B)** subgroup analyses for OS.

Supplementary Figure 3 Stratified analysis of PFS and OS across all treatment subgroups, categorized by prior exposure to PD-1 inhibitor–based therapy. **(A)** stratified analysis of PFS across all enrolled patients (154); **(B)** stratified analysis of OS across all enrolled patients (154); **(C)** stratified analysis of PFS across ITC and IC subgroups (130); **(D)** stratified analysis of OS across ITC and IC subgroups (130); **(E)** stratified analysis of PFS across combination arm (65); **(F)** stratified analysis of OS across combination arm (65); **(G)** stratified analysis of PFS across non-combination arm (89); **(H)** stratified analysis of OS across non-combination arm (89); **(I)** stratified analysis of PFS across ITC subgroup (56); **(J)** stratified analysis of OS across ITC subgroup (56); **(K)** stratified analysis of PFS across IC subgroup (74); **(L)** stratified analysis of OS across IC subgroup (74).
