# Supplementary material for: Efficacy and safety of apatinib or anlotinib combined with PD-1 inhibitors-based therapy as subsequent-line treatment for recurrent or metastatic nasopharyngeal carcinoma: a real-world retrospective study
Source: Front Oncol. 2025 Nov 6;15:1624286. doi: 10.3389/fonc.2025.1624286 (PMC12631233; doi:10.3389/fonc.2025.1624286)

Table S1 Baseline characteristics of the two treatment subgroups (ITC and IC). Abbreviations: BMI, body mass index; EBV, Epstein–Barr virus.

	**ITC group (N=56)**	**IC group (N=74)**	**Total（N=130）**	**P-value**
Age				
≤50	28 (50.0%)	30 (40.5%)	58 (44.6%)	0.561
>50	28 (50.0%)	44 (59.5%)	72 (55.4%)	
Gender				
Male	45 (80.4%)	56 (75.7%)	101 (77.7%)	0.817
Female	11 (19.6%)	18 (24.3%)	29 (22.3%)	
Smoking				
No	53 (94.6%)	65 (87.8%)	118 (90.8%)	0.414
Yes	3 (5.4%)	9 (12.2%)	12 (9.2%)	
Cancer family history				
No	55 (98.2%)	71 (95.9%)	126 (96.9%)	0.760
Yes	1 (1.8%)	3 (4.1%)	4 (3.1%)	
Underlying disease history				
No	43 (76.8%)	55 (74.3%)	98 (75.4%)	0.949
Yes	13 (23.2%)	19 (25.7%)	32 (24.6%)	
BMI				
<18	5 (8.9%)	6 (8.1%)	11 (8.5%)	0.883
18-24	32 (57.1%)	36 (48.6%)	68 (52.3%)	
>24	19 (33.9%)	32 (43.2%)	51 (39.2%)	
Disease status				
Recurrent	21 (37.5%)	20 (27.0%)	41 (31.5%)	0.445
Metastatic	35 (62.5%)	54 (73.0%)	89 (68.5%)	
Treatment lines				
2	41 (73.2%)	55 (74.3%)	96 (73.8%)	0.990
≥3	15 (26.8%)	19 (25.7%)	34 (26.2%)	
Liver metastasis				
No	40 (71.4%)	53 (71.6%)	93 (71.5%)	1.000
Yes	16 (28.6%)	21 (28.4%)	37 (28.5%)	
EBV DNA level				
Negative	24 (42.9%)	34 (45.9%)	58 (44.6%)	0.940
Positive	32 (57.1%)	40 (54.1%)	72 (55.4%)	
Number of metastatic sites				
0-3	30 (53.6%)	31 (41.9%)	61 (46.9%)	0.418

>3	26 (46.4%)	43 (58.1%)	69 (53.1%)	
Previous treatment				
Platinum based therapy	43 (76.8%)	57 (77.0%)	100 (76.9%)	0.999
PD-1-inhibitors based therapy	13 (23.2%)	17 (23.0%)	30 (23.1%)	
Local radiotherapy				
No	18 (32.1%)	35 (47.3%)	53 (40.8%)	0.220
Yes	38 (67.9%)	39 (52.7%)	77 (59.2%)	
Pathological classification (Squamous-cell carcinoma)				
Undiffrentiation	31 (55.4%)	33 (44.6%)	64 (49.2%)	0.478
Lowdifferentiation	25 (44.6%)	41 (55.4%)	66 (50.8%)	
Clinical stage				
III stage	10 (17.9%)	9 (12.2%)	19 (14.6%)	0.661
IV stage	46 (82.1%)	65 (87.8%)	111 (85.4%)	

Table S2 Tumor response in the two treatment groups (ITC and IC). Abbreviations: CR, complete response; PR, partial response; SD, stable disease; PD, progressive disease; ORR, objective response rate; DCR, disease control rate.

	**ITC group (N=56)**	**IC group(N=74)**	**Total (N=130)**	**P-Value**
CR	3 (5.4%)	1 (1.4%)	4 (3.1%)	
PR	24 (42.9%)	22 (29.7%)	46 (35.4%)	
SD	25 (44.6%))	37 (50.0%)	62 (47.7%)	
PD	4 (7.1%)	14 (18.9%)	18 (13.8%)	
ORR	27 (48.2%)	23 (31.1%)	50 (38.5%)	0.139
DCR	52 (92.9%)	60 (81.1%)	112 (86.2%)	0.157

Table S3 Univariate and multivariate Cox regression analysis of prognostic factors in ITC and IC subgroups (PFS). Abbreviations: HR: hazard ratio; CI: confidence interval; EBV: Epstein-Barr virus; PLT: Platelet; ALB: Albumin; LDH: Lactate dehydrogenase; PLR: platelet to lymphocyte ratio; NLR: neutrophil to lymphocyte ratio; LAR: Lactate dehydrogenase to Albumin ratio; PNI: Prognostic nutritional index.

	**Univariate analysis**		**Multivariable analysis**	
	**HR (95%CI)**	**P-value**	**HR (95%CI)**	**P-value**
Gender (female vs male)	1.023 (0.610-1.716)	0.931		
Age (>50 vs ≤50)	1.134 (0.736-1.746)	0.568		
EBV DNA level (positive vs negative)	1.741 (1.117-2.715)	**0.014**	1.600 (0.984-2.601)	0.058
Anemia (yes vs no)	1.483 (0.964-2.283)	0.073		
PLT (>110 vs ≤110)	0.593 (0.320-1.099)	0.097		
ALB (>41.3 vs ≤41.3)	0.520 (0.319-0.846)	**0.009**	1.018 (0.483-2.148)	0.962

LDH (>240 vs ≤240)	2.355 (1.394-3.980)	**0.001**	3.698 (1.523-8.979)	**0.004**
PLR (>94.37 vs ≤94.37)	0.512 (0.300-0.873)	**0.014**	0.616 (0.327-1.163)	0.135
NLR (>5.5 vs ≤5.5)	1.684 (1.060-2.675)	**0.027**	1.143 (0.635-2.055)	0.656
LAR (>5.43 vs ≤5.43)	2.678 (1.608-4.460)	**<0.001**	0.469 (0.179-1.288)	0.123
PNI (>47.2 vs ≤47.2)	0.423 (0.269-0.666)	**<0.001**	0.286 (0.135-0.605)	**0.001**
Treatment lines (≥3 vs 2)	1.344 (0.851-2.124)	0.205		
Distant metastasis (Yes vs No)	1.247 (0.785-1.981)	0.350		
Liver metastasis (Yes vs No)	1.522 (0.966-2.397)	0.070		
Bone metastasis (Yes vs No)	1.346 (0.871-2.079)	0.181		
Lung metastasis (Yes vs No)	0.945 (0.584-1.528)	0.817		
Number of metastatic sites (>3 vs 0-3)	1.902 (1.222-2.959)	**0.004**	1.767 (1.055-2.958)	**0.031**
Treatment cycles (>6 vs 2-6)	0.402 (0.257-0.630)	**<0.001**	0.294 (0.171-0.506)	**<0.001**
Combination treatment (ITC vs IC)	0.372 (0.232-0.595)	**<0.001**	0.387 (0.232-0.645)	**<0.001**
Local radiotherapy (Yes vs No)	0.820 (0.531-1.267)	0.372		
Previous treatment (PD-1 inhibitors based therapy vs Platinum based therapy)	1.844 (1.128-3.014)	**0.015**	1.971 (1.120-3.469)	**0.019**
Pathological classification (Undiffrentiation vs Lowdifferentiation)	0.961 (0.619-1.490)	0.858		
Clinical stage (IV vs III)	1.544 (0.795-3.000)	0.199		

Table S4 Univariate and multivariate Cox regression analysis of prognostic factors in ITC and IC subgroups (OS). Abbreviations: HR: hazard ratio; CI: confidence interval; EBV: Epstein-Barr virus; PLT: Platelet; ALB: Albumin; LDH: Lactate dehydrogenase; PLR: platelet to lymphocyte ratio; NLR: neutrophil to lymphocyte ratio; LAR: Lactate dehydrogenase to Albumin ratio; PNI: Prognostic nutritional index.

	**Univariate analysis**		**Multivariable analysis**	
	**HR (95%CI)**	P-value	**HR (95%CI)**	**P-value**
Gender (female vs male)	1.257 (0.672-2.352)	0.474		
Age (>50 vs ≤50)	1.221 (0.706-2.112)	0.475		
EBV DNA level (positive vs negative)	2.440 (1.355-4.393)	**<0.001**	1.461 (0.741-2.879)	0.274
Anemia (yes vs no)	1.779 (1.038-3.050)	**0.036**	0.762 (0.375-1.546)	0.451
PLT (>110 vs ≤110)	0.672 (0.300-1.505)	0.334		
ALB (>41.3 vs ≤41.3)	0.437 (0.243-0.787)	**0.006**	1.168 (0.453-3.013)	0.748
LDH (>240 vs ≤240)	1.863 (0.957-3.624)	0.067		
PLR (>94.37 vs ≤94.37)	0.528 (0.271-1.028)	0.060		
NLR (>5.5 vs ≤5.5)	1.865 (1.062-3.276)	**0.030**	0.803 (0.392-1.647)	0.550
LAR (>5.43 vs ≤5.43)	3.153 (1.743-5.072)	**<0.001**	1.406 (0.611-3.238)	0.423
PNI (>47.2 vs ≤47.2)	0.304 (0.176-0.524)	**<0.001**	0.169 (0.063-0.453)	**<0.001**
Treatment lines (≥3 vs 2)	1.321 (0.741-2.356)	0.345		
Distant metastasis (Yes vs No)	1.382 (0.750-2.548)	0.300		

Liver metastasis (Yes vs No)	1.990 (1.139-3.476)	**0.016**	1.306 (0.623-2.736)	0.479
Bone metastasis (Yes vs No)	1.201 (0.694-2.077)	0.513		
Lung metastasis (Yes vs No)	1.306 (0.739-2.308)	0.358		
Number of metastatic sites (>3 vs 0-3)	2.349 (1.304-4.232)	**0.004**	1.675 (0.844-3.326)	0.141
Treatment cycles (>6 vs 2-6)	0.328 (0.182-0.593)	**<0.001**	0.220 (0.109-0.444)	**<0.001**
Combination treatment (ITC vs IC)	0.494 (0.274-0.889)	**0.019**	0.815 (0.425-1.561)	0.537
Local radiotherapy (Yes vs No)	0.653 (0.380-1.121)	0.122		
Previous treatment (PD-1 inhibitors based therapy vs Platinum based therapy)	1.985 (1.069-3.687)	**0.030**	2.971 (1.452-6.081)	**0.003**
Pathological classification (Undiffrentiation vs Lowdifferentiation)	0.957 (0.552-1.660)	0.876		
Clinical stage (IV vs III)	1.286 (0.579-2.852)	0.537		

Table S5 Treatment-related adverse events in ITC and IC subgroups. ALT: alanine aminotransferase; AST: aspartate aminotransferase

	All grades			≥3 grade		
	ITC group(N=57)	IC group (N=74)	P-value	ITC group(N=56)	IC group(N=74)	P-value
Fatigue	24 (42.9%)	24 (32.4%)	0.475	7 (12.5%)	5 (6.8%)	0.534
Nausea	28 (50.0%)	31 (41.9%)	0.655	7 (12.5%)	5(6.8%)	0.534
Anemia	46 (82.1%)	62 (83.8%)	0.970	2 (3.6%)	8 (10.8%)	0.308
Leukopenia	40 (71.4%)	52 (70.3%)	0.990	13 (23.2%)	20 (27.0%)	0.885
Thrombocytopenia	32 (57.1%)	42 (56.8%)	0.999	3 (5.4%)	12 (16.2%)	0.159
Hypertension	19 (33.9%)	12 (16.2%)	0.064	3 (5.4%)	2 (2.7%)	0.738
ALT elevation	23 (41.1%)	28 (37.8%)	0.932	3 (5.4%)	2 (2.7%)	0.738
AST elevation	16 (28.6%)	26 (35.1%)	0.731	1 (1.8%)	2 (2.7%)	0.942
Hypoalbuminemia	20 (35.7%)	18 (24.3%)	0.368	0	0	
Rash	4 (7.1%)	2 (2.7%)	0.490	2 (3.6%)	0	0.261
Pneumonia	3 (5.4%)	2 (2.7%)	0.738	0	1 (1.4%)	0.683
Epistaxis	3 (5.4%)	4 (5.4%)	1.000	3 (5.4%)	0	0.131
NP necrosis	4 (7.1%)	4 (5.4%)	0.920	1 (1.8%)	1 (1.4%)	0.980
Cough	5 (8.9%)	2 (2.7%)	0.297	0	0	
Hypothyroidism	17 (30.4%)	34 (45.9%)	0.197	4 (7.1%)	7 (9.5%)	0.895
Total	54 (96.4%)	70 (94.6%)	0.885	22 (39.3%)	24 (32.4%)	0.721

Figure S1 The optimal cut-off values for hematological markers were determined using the surv_cutpoint function from the survminer package in R, which identifies the most statistically significant threshold based on maximally selected rank statistics. (PLT: Platelet; ALB: Albumin; LDH: Lactate dehydrogenase; PLR: platelet to lymphpcyte ratio; NLR: neutrophil to lymphocyte ratio; LAR: Lactate dehydrogenase to Albumin ratio; PNI: Prognostic nutritional index.)

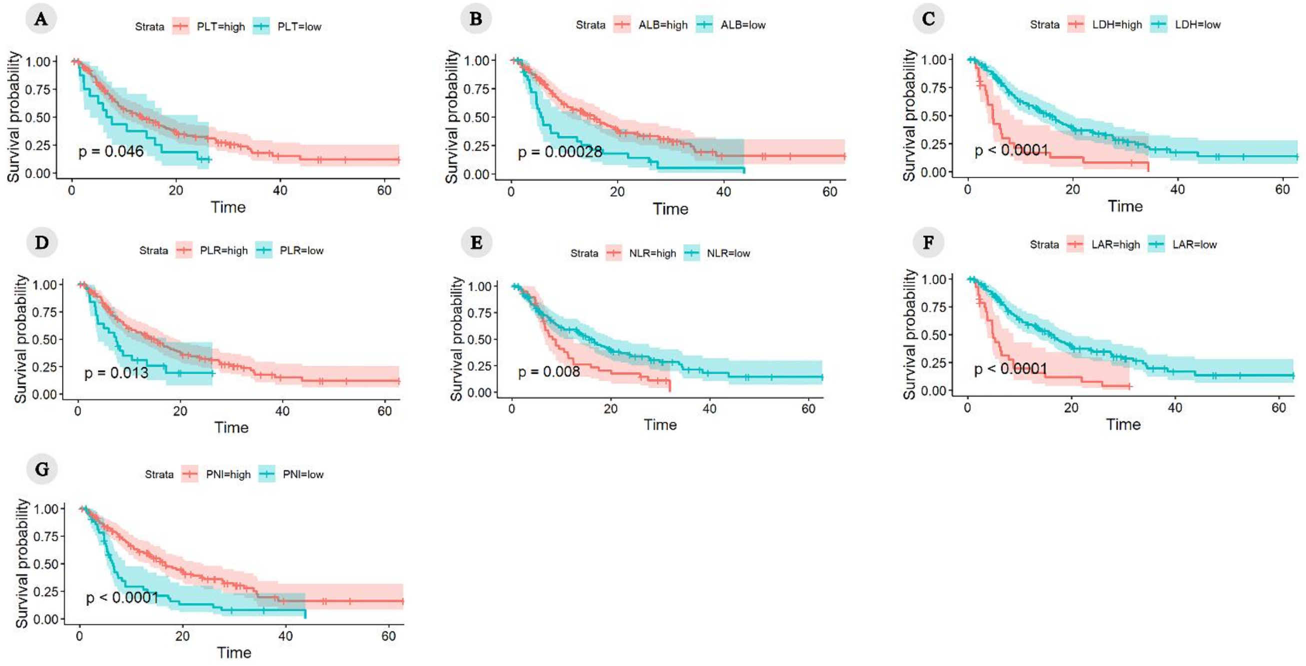

Figure S2 Forest plot of subgroup analyses (ITC and IC). Abbreviations: HR: hazard ratio; CI: confidence interval; EBV: Epstein-Barr virus; PNI: Prognostic nutritional index. Figure S2A：subgroup analyses for PFS; Figure S2B: subgroup analyses for OS.

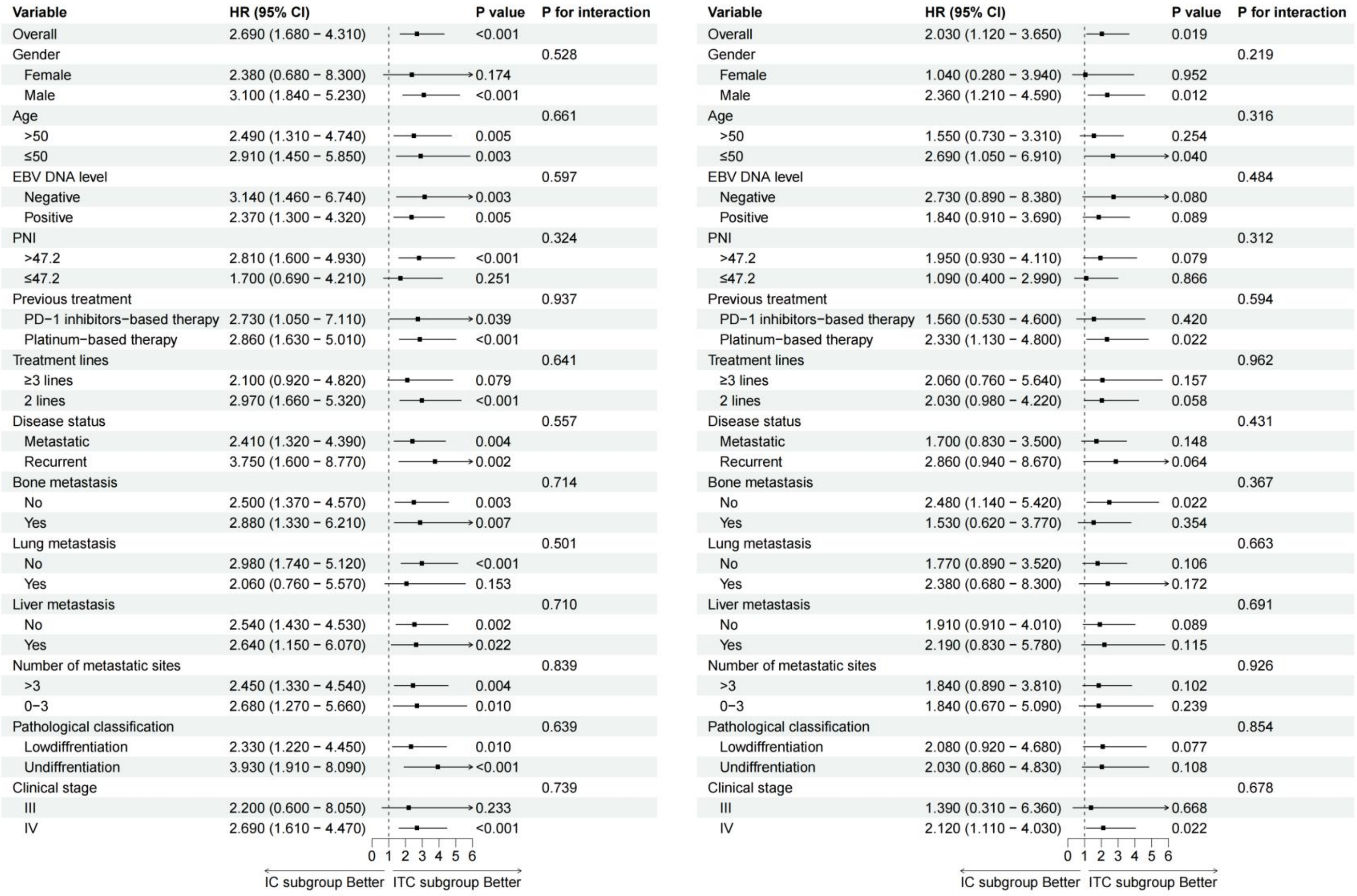

Figure S3 Stratified analysis of PFS and OS across all treatment subgroups, categorized by prior exposure to PD-1 inhibitor - based therapy.

Figure S3A: stratified analysis of PFS across all enrolled patients (154);

Figure S3B: stratified analysis of OS across all enrolled patients (154);

Figure S3C: stratified analysis of PFS across ITC and IC subgroups (130);

Figure S3D: stratified analysis of OS across ITC and IC subgroups (130);

Figure S3E: stratified analysis of PFS across combination arm (65);

Figure S3F: stratified analysis of OS across combination arm (65);

Figure S3G: stratified analysis of PFS across non-combination arm (89);

Figure S3H: stratified analysis of OS across non-combination arm (89);

Figure S3I: stratified analysis of PFS across ITC subgroup (56);

Figure S3J: stratified analysis of OS across ITC subgroup (56);

Figure S3K: stratified analysis of PFS across IC subgroup (74);

Figure S3L: stratified analysis of OS across IC subgroup (74);

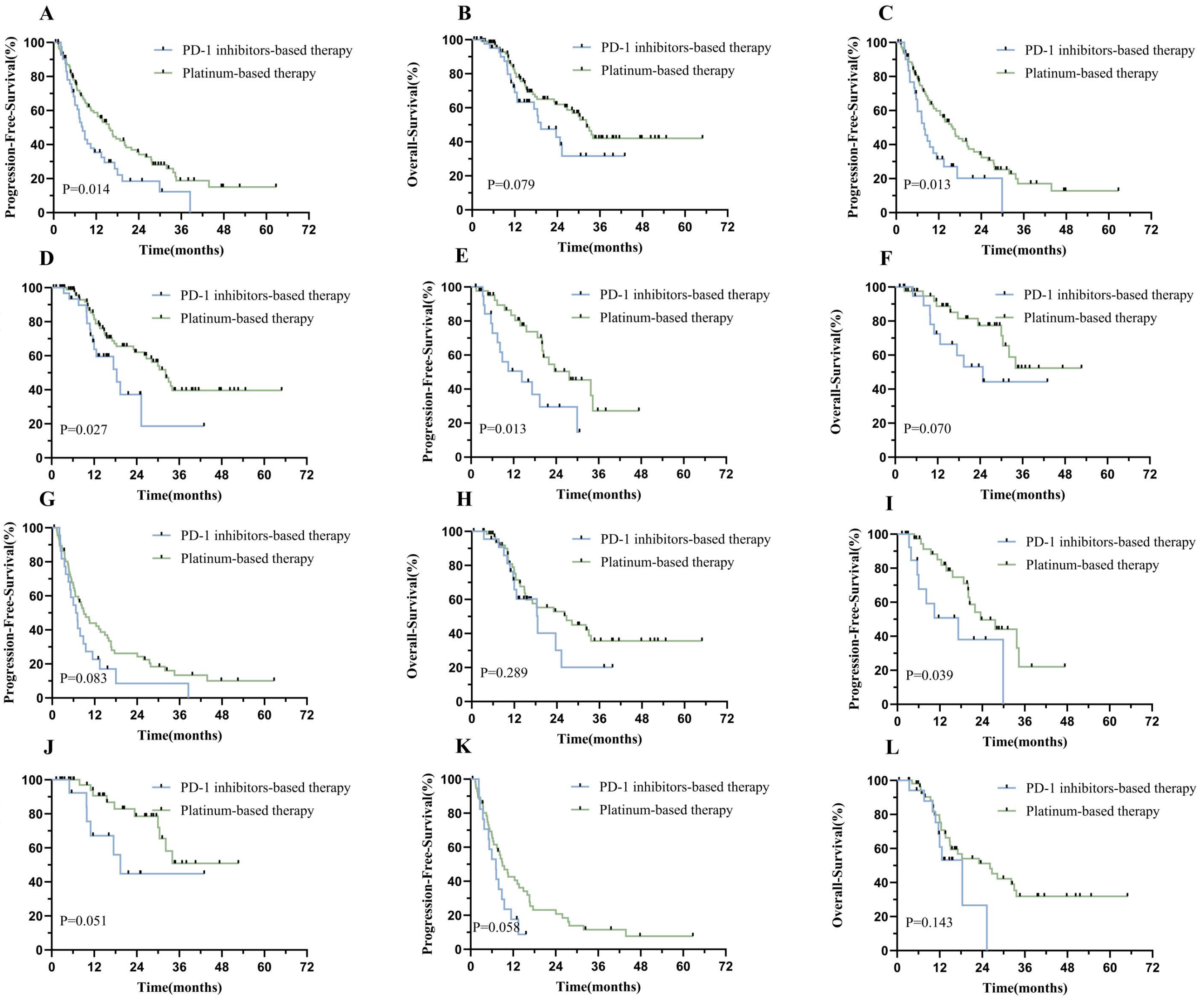

Supplement: Supplementary Table 1 — Baseline characteristics of the two treatment subgroups (ITC and IC). BMI, body mass index; EBV, Epstein–Barr virus. [file DataSheet1.pdf]
